# The effect of hydroalcoholic extract of *Coriandrum sativum* on rat appetite

**Published:** 2013

**Authors:** Mohsen Nematy, Maryam Kamgar, Seyed Mohammad Reza Mohajeri, Seyed Amir Tabatabaei Zadeh, Mohammad Reza Jomezadeh, Omid Akbarieh Hasani, Najmeh Kamali, Shohreh Vojouhi, Sara Baghban, Azita Aghaei, Mohammad Soukhtanloo, Mahmoud Hosseini, Zahra Gholamnezhad, Hassan Rakhshandeh, Abdolreza Norouzy, Habibollah Esmaily, Majid Ghayour-Mobarhan, Michael Patterson

**Affiliations:** 1 *Department of Nutrition, Biochemistry of Nutrition, Endoscopic & Minimally Invasive Surgery, and Cancer Research Centers, School of Medicine, Mashhad University of Medical Sciences, Mashhad, I. R. Iran*; 2 *Department of Nutrition, School of Medicine, Mashhad University of Medical Sciences, Mashhad, I. R. Iran*; 3 *Medical Student, School of Medicine, Mashhad University of Medical Sciences, Mashhad, I. R. Iran*; 4 *Department of Physiology, School of Medicine, Mashhad University of Medical Sciences, Mashhad, I. R. Iran*; 5 *Pharmacological Research Center of Medicinal Plants, School of Medicine, Mashhad University of Medical Sciences, Mashhad, I. R. Iran*; 6 *Health Sciences Research Center, Department of Biostatistics and Epidemiology, School of Health, Mashhad University of Medical Sciences, Mashhad, I. R. Iran*; 7 *Cardiovascular Research Center and Biochemistry of Nutrition Research center, School of Medicine, Mashhad University of Medical Sciences, Mashhad, I. R. Iran*; 8 *Nutrition & Dietetics Research Group, Hammersmith Hospital, Imperial College London, Du Cane Road, London W12 0HS UK*

**Keywords:** *Coriandrum sativum*, Appetite, Energy intake, Rat

## Abstract

**Objective:** Losing weight in consequence of appetite loss can be a sign of a serious underlying condition. Currently, the most widely prescribed medication for anorexia is cyproheptadine hydrochloride. However, the clinical use of cyproheptadine hydrochloride is limited by its side effects. In Iranian traditional medicine, *Coriandrum sativum* stimulates the appetite. Therefore, the effect of *Coriandrum sativum* (coriander) hydroalcoholic extract was investigated on food intake in rats.

**Material and Methods:** Thirty male Wistar rats were randomly divided into five groups. Two control groups were used, one group received 0.5 ml water per day (vehicle group), and another group did not receive anything (control group). The other 3 groups were daily treated by 50, 100 or 150 mg/kg of coriander for 7 days, respectively. The daily amount of the food eaten by each rat was measured for 10 days. The amount of energy intake of each rat was also calculated for 7 days during the intervention. The difference in energy intake was calculated and compared between groups.

**Result:** There was no significant change in energy intake between control and vehicle groups. The change in energy intake after treatment by 100 and 150 mg/kg of the extract was significantly higher than other groups (p=0.030 and p=0.007)

**Conclusion:** This study indicated that coriander had positive effects on appetite of rats. Future studies are needed to evaluate the mechanisms of the effects of this plant on appetite.

## Introduction

Most people have experienced a temporary loss of appetite at some time. This is rarely a worrisome symptom unless it lasts for more than a day or two. It can also be a sign of a serious underlying condition, such as depression or cancer cachexia. It also commonly occurs during a sudden illness, such as an infection (Chapman and Nelson, 1994 ▶). When loss of appetite continues for a long time, a person is at risk for malnutrition and micronutrients deficiencies (Brownie 2006 ▶; Chandra et al., 1991 ▶). 

Appetite stimulants have been used to help overcome decreased appetite. A few examples of stimulants are megestrol acetate (MA), cyproheptadine hydrochloride (CH), cannabinoids, hydrazine sulfate, anabolic hormones, and growth hormone. Currently, the most widely prescribed medication for anorexia is cyproheptadine hydrochloride (Kardinal et al., 1990 ▶). However, the clinical uses of cyproheptadine hydrochloride is limited by its side effects such as somnolence, excitation, hallucinations, ataxia, tachycardia, and muscle twitching, and occasionally gastric pain, dry mucous surfaces, mydriasis, and rubeosis of the face (Von Mühlendahl and Krienke 1978 ▶). Some contraindications of using these medications are lactation, pregnancy, and hypersensitivity (Anon 2009). MA, has substantial side effects and may not be suitable for prolonged use (Homnick et al., 2005 ▶).

Plants not only provide food and shelter but also serve humanity by preventing and curing different ailments. Herbs and spices have always been useful to cure diseases (Tipu et al., 2006 ▶). The practice of herbal medicine dates back to the very earliest period of known human history. There is evidence of herbs having been used in the treatment of diseases and for revitalizing body system in almost all ancient civilizations, the Egyptian, the Chinese and even Greek and Roman civilizations (Aftab and Sial 1999 ▶). Currently, there is a high interest in recognition of new potential medications that have minimal side effects which can be used in medicine and the food industry (Srivastava et al., 1993 ▶). 

*Coriandrum sativum* (Coriander) is an herb in the family Apiaceae. Although, all parts of the plant are edible, its fresh leaves and dried seeds are most frequently used (Benzie and Wachtel-Galor 2011 ▶), which contain an essential oil and the monoterpenoid-linalool (Eikani et al., 2007 ▶). In traditional medicine, *Coriandrum sativum* was used in the preparation of many household medicines to relief anxiety and insomnia, and may have potential sedative, hypotensive, and muscle relaxant effects. Its beneficial effects in bed cold, seasonal fever, nausea, vomiting, and stomach disorders have been suggested. It has also been used as a drug for rheumatoid disorders, pain in the joints, and against worms (Emamghoreishi et al., 2005 ▶; Medhin et al., 1986 ▶; Rajeshwari and Andallu, 2011 ▶). Several animal studies provide evidence that coriander seeds can promote the hepatic antioxidant system (Anilakumar et al., 2001 ▶; Aruna et al., 2006 ▶). Study of Emamghoreishi and colleagues suggests that the aqueous extract of *Coriandrum sativum* seed has anxiolytic effect and may have potential sedative and muscle relaxant effects (Emamghoreishi, Khasaki & Aazam 2005 ▶). 

Coriander is digestive and appetite stimulant in ancient medicine (VanNostrand and Sarles 2002 ▶). In another study, (Banerjee et al., 1994 ▶) observed roughly a doubling in glutathione S-transferase (GST) protein activity in Swiss albino mice that were provided with diets containing coriander oil. Moreover, few studies suggest that coriander may have anticancer properties (Esiyok et al., 2004 ▶). Many of healing properties of *Coriandrum sativum* can be attributed to its exceptional phytonutrients and hence, it is often referred to as the store house for bioactive compounds (Rajeshwari and Andallu, 2011 ▶). However, no pharmacological or medical studies have evaluated the effects of *Coriandrum sativum* on appetite. In Iranian traditional medicine, coriander is used as an orexigenic agent, so in this research for the first time, we investigated the effect of the extract of this plant on food intake in rats.

## Material and Method

**Animals**

Thirty male Wistar rats with age of 8 weeks and 200-220 g weight were purchased from animal house of Mashhad University of Medical Sciences, Mashhad, Iran. Laboratory temperature was 22±1 °C with 12 hours light/dark cycle, and all rats had access to the source of food without any limitation. 

**Foods**

Laboratory animal food was provided from Javaneh Khorasan Company that includes 21% protein and 6.5 – 7% fat. Every 1000 g of food contain 2750 Kilocalories energy. 

**Preparation of hydroalcoholic extract**

*Coriandrum sativum* was provided by University herbarium. Pharmacologic parts of coriander are seed, leaf and stem. These parts were dried at room temperature in shadow, and then powdered. Maceration extraction method was used for extraction. Powder was inserted in closed can and solvent (70% alcohol) was added. Then the solution was kept in the can for 2-4 days and mixed one or two times a day. The solution was filtered and liquid was separated with rotary evaporator. After that, the extract was dried in 40 °C oven. The extract was dissolved in water before gavage every day.

**Experimental design**

Thirty rats were located to five groups randomly. 1- control: the animals in this group were without any treatment, 2- vehicle: the animals received 0.5 ml water per day instead of the extract, 3, 4, and 5: three types of solution with different concentrations of *Coriandrum sativum* were provided to the animals (50, 100, and 150 mg/kg of the hydroalcoholic extract, respectively). The daily amount of food eaten by each rat was measured for 10 days before intervention to calculate the used calories in 24 hours. The animals of all groups were gavaged with the extract or water. The animals of control group didn’t receive anything. The daily energy intake of each rat was calculated for 7 days after the treatment.

This study was approved by Ethics Committee of Vice Chancellor for Research of Mashhad University of Medical Sciences (MUMS), Mashhad, Iran 

**Statistical** **analysis**

All data were analyzed using ANOVA and paired samples t-test. P-values less than 0.05 were considered to be statistically significant

## Results

The mean energy intake before intervention was not different between groups (Table 1). Treatment of the animals with 50 mg/ kg of the extract did not increase the energy intake significantly. In the animals which were treated with 100 mg/ kg of the extract, the daily energy intake was 58.450±2.230 Kcal which was significantly higher compared with the amount of energy intake before the treatment (p=0.030). The daily energy intake in the animals treated with 150 mg/ kg of the extract was also increased compared with the before treatment (56.416±1.800 Kcal; vs. 52.483±1.543, p=0.007). In addition, comparison of mean energy intake changes along the study (during intervention – before intervention) between first case group and vehicle group, between first case group and control group, between second case group and vehicle group, and also between second case group and control group did not show any significant change (p=0.997, p=0.999, p=0.555, p=0.690, respectively) (Table 2). However, assessment of mean energy intake changes along the study between third case group and vehicle group, and also between third group and control group showed significant change (p=0.002, p=0.002, respectively). 

**Table 1:** Mean energy intake in 24 hours before and during intervention in five groups of rats in the study

Group	Before intervention (Kcal)*	During intervention (Kcal)**	Change (Kcal)	p-value
First case group (received 50 mg/kg coriander solution)	52.066±0.600	53.200±0.727	1.100±1.882	0.496
Second case group (received 100 mg/kg coriander solution)	53.366±0.904	58.450±2.230	3.280±1.640	0.030
Third case group (received 150 mg/kg coriander solution)	52.483±1.543	56.416±1.800	4.360±1.700	0.007
Control group	52.650±1.380	52.916±1.360	0.350±0.615	0.523
Vehicle group	52.783±1.370	53.133±1.250	0.266±0.952	0.222

* Mean±SEM (KCal),

** Paired sample t-test

**Table 2:** Comparison of mean energy intake in different rats groups

	Vehicle group (p-value)*	Control group (p-value)
First case group	0.997	0.999
Second case group	0.555	0.690
Third case group	0.002	0.002

* Tukey-Kramer Test

## Discussion

In Iranian traditional medicine there are many medical plants that increase the appetite and probably have fewer side effects as well fewer costs than chemical drugs. One of these medical plants is *Coriandrum sativum*. In traditional medicine, the beneficial effects of* Coriandrum sativum* L. has been suggested for a number of medical problems such as dyspeptic complaints, loss of appetite, convulsion, insomnia, and anxiety (Mir Heidar 1992 ▶; Zargari 1991 ▶).

In the present study, 100 and 150 mg/kg of hydroalcoholic extract of coriander increased appetite in rats. The results of the present study confirmed the traditional believe regarding the beneficial effects of *Coriandrum sativum* for increasing the appetite (Avecina A, 1992 ▶). In contrast to these findings, Gray et al. reported that *Coriandrum sativum* had no significant effect on food intake in diabetic mice (Gray and Flatt, 1999 ▶).

The components of *C. sativum* include essential oil, glucose, fructose and sucrose, alkaloids, flavones, resins, tannins, anthraquinones, beta-sitosterol, beta-sitosteroline, fixed oils, linalool, and carotenoids (Trease and Evans, 1996 ▶).

Based on the results of the present study, it is impossible to conclude about the effective compound(s) of the extract or mechanism(s) responsible for the effect of the extract. Several endogenous substances have been related to satiety–hunger cycle disorders. TNF-α has been recognized as having an anorexigenic effect under different conditions. TNF-α is associated with anorexia in wasting syndromes (Beutler and Cerami 1986 ▶). Coriander seeds have flavonoid compounds including quercetin and routine (Blumenthal et al., 2000 ▶). Some researchers have shown that these compounds decrease TNF-α, so it might be suggested that the extract of coriander stimulate appetite with decreasing TNF-α. Of course coriander may stimulate appetite with other probable mechanisms such as gastrointestinal hormones. No more evidence was found about relation of chemical composition of *Coriandrum sativum* and anorexia. More studies are needed to find the mechanism of coriander extract for increasing appetite. Measuring appetite regulating hormones are also important in evaluating the extract mechanism(s). It has been reported that *Coriandrum sativum* increases insulin secretion in vitro (Gray & Flatt 1999 ▶). Regarding this effect and antidiabetic actions of the plant, it is conceivable that the effects of extract on food intake which was seen in the present study may be in part be due to this mechanism.

Linalool is the main ingredient in coriander (Usta et al., 2009 ▶). Moreover, it has been reported that linalool reduces the plasma glycerol level and increases food intake and body weight (Shen et al., 2005 ▶). Therefore, the increase in food intake which was seen in the present study may at least be in part due to this component.

Regarding the effects of *Coriandrum sativum* on central nervous system and considering some of its effects such as antianxiety, antidepressant, relaxant, and hypnotic effects which have been previously reported (Emamghoreishi, Khasaki, & Aazam 2005 ▶; Rakhshandeh et al., 2011 ▶; Zargar-Nattaj et al., 2011 ▶), the role of neurotransmitter systems such as serotonergic, dopaminergic and opioids system in mediating the possible effects of the plant on appetite which was seen in the present study should not be ignored.

This study indicated that coriander had positive effect on appetite of rats. Future studies evaluating effects of coriander on human appetite are warranted.
